# Trends in DDT and pyrethroid resistance in *Anopheles gambiae *s.s. populations from urban and agro-industrial settings in southern Cameroon

**DOI:** 10.1186/1471-2334-9-163

**Published:** 2009-09-30

**Authors:** Philippe Nwane, Josiane Etang, Mouhamadou Chouaibou, Jean Claude Toto, Clément Kerah-Hinzoumbé, Rémy Mimpfoundi, Herman Parfait Awono-Ambene, Frédéric Simard

**Affiliations:** 1 Organisation de Coordination pour la lutte contre les Endémies en Afrique Centrale, Yaoundé, Cameroun; 2 Université de Yaoundé I, Yaoundé, Cameroun; 3 Faculty of Medicine and Pharmaceutical Sciences, University of Douala, Cameroun; 4 Programme National de Lutte contre le Paludisme, N'Djamena, Tchad; 5 Institut de Recherche pour le Développement (IRD), UR016, Bobo-Dioulasso, Burkina Faso

## Abstract

**Background:**

Pyrethroid insecticides are widely used for insect pest control in Cameroon. In certain insect species, particularly the malaria vector *Anopheles gambiae*, resistance to this class of insecticides is a source of great concern and needs to be monitored in order to sustain the efficacy of vector control operations in the fields. This study highlights trends in DDT and pyrethroid resistance in wild *An. gambiae *populations from South Cameroon.

**Methods:**

Mosquitoes were collected between 2001 and 2007 in four sites in South Cameroon, where insecticides are used for agricultural or personal protection purposes. Insecticide use was documented in each site by interviewing residents. Batches of 2-4 days old adult female mosquitoes reared from larval collections were tested for susceptibility to DDT, permethrin and deltamethrin using standard WHO procedures. Control, dead and survivors mosquitoes from bioassays were identified by PCR-RFLP and characterized for the *kdr *mutations using either the AS-PCR or the HOLA method.

**Results:**

Four chemical insecticide groups were cited in the study sites: organochlorines, organophosphates, carbamates and pyrethroids. These chemicals were used for personal, crop or wood protection. In the four *An. gambiae *populations tested, significant variation in resistance levels, molecular forms composition and *kdr *frequencies were recorded in the time span of the study. Increases in DDT and pyrethroid resistance, as observed in most areas, were generally associated with an increase in the relative frequency of the S molecular form carrying the *kdr *mutations at higher frequencies. In Mangoum, however, where only the S form was present, a significant increase in the frequency of *kdr *alleles between 2003 to 2007 diverged with a decrease of the level of resistance to DDT and pyrethroids. Analyses of the *kdr *frequencies in dead and surviving mosquitoes showed partial correlation between the *kdr *genotypes and resistance phenotypes, suggesting that the *kdr *mechanism may act with certain co-factors to be identified.

**Conclusion:**

These results demonstrate the ongoing spread of *kdr *alleles in *An. gambiae *in Central Africa. The rapid evolution of insecticide resistance in this highly dynamic and genetically polymorphic species remains a challenge for its control.

## Background

Agriculture and timber production are the main components of the economical activities in most equatorial African countries. These economic activities require intensive use of pesticides including insecticides belonging to the four main chemical groups used in public health: organochlorines, carbamates, organophosphates and pyrethroids. In Cameroon, organochlorines such as DDT have been used extensively for both vector control and agricultural purposes in the southern areas of the country, especially during the 1950s malaria eradication campaign [1-3]. However, these compounds have progressively been replaced by alternative more specific and less toxic chemicals, in part because of the emergence of insecticide resistance in the target species [1,4]. Nowadays, pyrethroids are largely recommended because of their high effectiveness and strong excito-repellent effect on insects, as well as low mammalian toxicity [5-8]. However, the extensive exposure of insect pests to these insecticides has already selected resistance in wild insect populations [9,10]. To date, more than 500 species of insects and mites have been reported to develop resistance to about 300 insecticide compounds [11-13]. Among these species, 56% are crop pests, 39% are arthropods of medical or veterinary importance and 5% are beneficial species [14].

The emergence of insect resistance to insecticides may decrease crop productivity [15] or reduce the effectiveness of insecticide treated nets or indoor residual spraying [13,16]. Resistance management is therefore a major challenge for vector control programmes in countries where vector-borne diseases are endemic and subsistence remains a burden to the communities. Malaria is the most devastating of all vector-borne diseases; it impedes on economic development not only by causing premature death but also through lost/diminished productivity, absenteeism, huge medical cost, and negative impact on fertility, population growth, and country's savings and investments [17].

Several insecticide resistance mechanisms have been reported in different classes of insects of medical and economic importance, particularly among major malaria vectors belonging to the *Anopheles gambiae *complex [18-21]. These mechanisms include enhanced detoxification of insecticides through increased enzymatic activities of esterases, gluthatione S-transferases and cytochrome P_450 _monooxygenases, mainly due to their overproduction as a result of gene amplification [22,23] and/or gene regulation [24-26]. Point mutations at the target sites of insecticides, decreasing the affinity of the insecticide to its receptor, constitute the second major and most widespread mechanism by which insects are able to resist insecticides [27]. Two mutations at amino acid position 1014 of the voltage-gated sodium channel, changing either a Leucine residue to a Phenylalanine (L1014F) [28], or a Leucine to a Serine (L1014S) [29] have been identified in *Anopheles gambiae *and confer knockdown resistance (*kdr*) to DDT and pyrethroid insecticides. On the other hand, organophosphates and carbamates are acetylcholinesterase inhibitors; structural changes in this enzyme in *Drosophila melanogaster*, *Musca domestica *and *An. gambiae *were reported to cause resistance to these insecticides [30-33].

In Cameroon, cross-sectional surveys of *An. gambiae *s.l. susceptibility to insecticides have been carried out since 1997. Metabolic resistance was suspected in some *An. gambiae *populations in 2003 and confirmed in 2007 [34,35]. More recently, the presence of both *kdr *mutations and their relationship with phenotypic resistance to pyrethroids and DDT were reported in Cameroon [36,37]. Here, we explore trends in insecticide resistance in wild *An. gambiae *populations from Southern Cameroon, through the longitudinal follow-up of insecticide susceptibility levels in four sites where insecticides are used extensively for agricultural or personal protection purposes.

## Methods

### Study sites and insecticide usage

**Figure 1:**
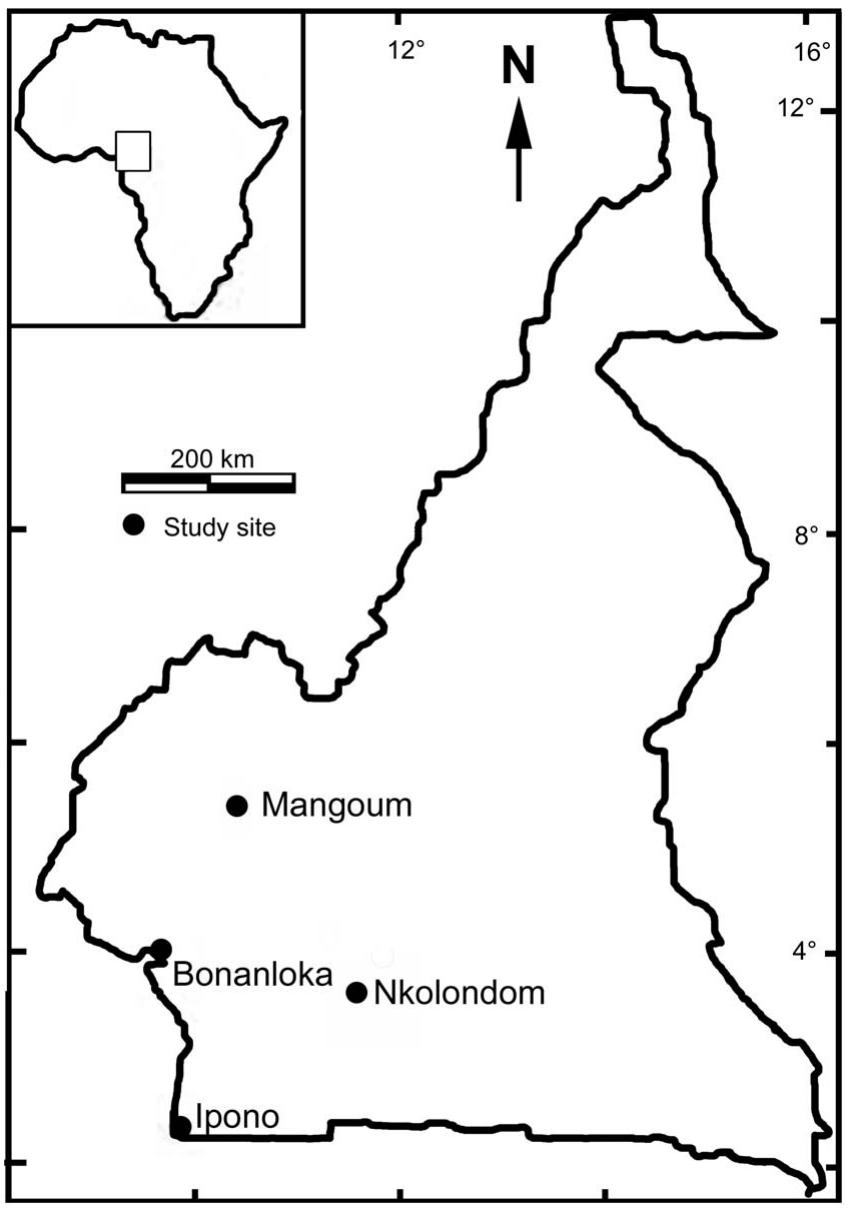
**Map of Cameroon showing study sites**.

The study was conducted in 4 localities (Figure 1) of Southern Cameroon that are characterized by high insecticide usage for agro-industry or personal protection: (1) Ipono district (2°22'N, 9°50'E), an area of intensive forest exploitation and timber storage, with agricultural activities limited to the households' supplies, (2) Bonanloka district (4°03'N, 9°43'E), a highly urbanized area in the centre of Douala, the major economic city in Cameroon, where inhabitants use insecticides against arthropod nuisance, (3) Nkolondom district (3°51'N, 11°30'E), a market gardening area located in the outskirts of Yaoundé, the capital city of Cameroon, and (4) Mangoum district (5°31'N, 10°37'E), a locality with extensive manual and mechanized agricultural settings producing spices, vegetables and cereals. These sites are among the places in Cameroon where agricultural and forest exploitation practices are sustained by pesticide application.

These localities are geographically located in the southern region of the country under the equatorial climate, with average yearly rainfall above 1,500 mm spread out over 4 seasons: 2 dry seasons (December-February and July-August) and 2 rainy seasons (March-June and September-November). However, noticeable variations of these climatic trends are observed in Mangoum located in the western mountain grassland characterized by one dry season between November and February, and one rainy season between March and October [38]. Mosquito larvae were collected during the rainy seasons in 2001, 2003, 2005 and 2007.

During mosquitoes sampling in 2003, a qualitative survey was conducted in each site with the aim to document the most widely used insecticide compounds and agricultural practices. Data concerning insecticide usage in public health, personal protection and agro-industry were collected by filling a standardized questionnaire. Questions asked to local inhabitants, gardeners or owners of timber yards were mainly focused on: (1) insecticides used (trade names/active ingredients), (2) cultivated crops and their treatment cycles, (3) operational dosages during crop treatments and (4) exploited surface for plant farming or wood storage.

### Mosquito collections and bioassays

Anopheline larvae were collected from a wide range of breeding sites, representative of the diversity of the mosquito population in each study site. Larvae were collected from puddles, flooded furrows, shallow wells, tire tracks, ponds and marshes. In each location, larval collections were performed in at least 20 breeding sites in which an average of 30 larvae (all instars) per breeding site were collected and reared locally to adults, and fed with a 10% sucrose solution. Upon emergence, mosquitoes were sexed and morphologically identified using morphological identification keys [39,40]. Only females *An. gambiae *s.l. were used for insecticide resistance monitoring. Susceptibility tests were carried out using WHO insecticide susceptibility test-kits and standard protocol for adults [41]. Impregnated filter papers (4% DDT, 0.75% permethrin and 0.05% deltamethrin) were provided by the Vector Control and Research Unit, University Sains Malaysia (Penang, Malaysia). Bioassays were performed at a temperature ranging from 25 to 28°C on 2-4-days old females. For each test, 80-100 mosquitoes separated into 4 batches were exposed to impregnated filter papers, while a batch of 20-25 mosquitoes served as control. The number of knockdown mosquitoes was recorded at 5 min intervals during 1 h exposure and mortality was determined 24 h post-exposure. At each sampling period, bioassays were concomitantly carried out with the Kisumu strain of *An. gambiae *maintained in the Laboratoire de Recherche sur le Paludisme at OCEAC (Yaounde, Cameroon) and used as the reference susceptible strain. Assayed samples were preserved individually on dessiccant (silica gel) and stored at -20°C for further analysis.

### Molecular identification and *kdr *genotyping

Upon completion of the susceptibility tests and recording of the individual phenotypes, random samples of mosquitoes from bioassays batches at each study period e.g. control, dead and surviving specimens were subjected to DNA extraction [42]. Specimens were identified to species and molecular form by PCR-RFLP [43]. Their genotype at the *kdr *locus was determined using either the Allele-Specific PCR (AS-PCR) [28] in samples collected in 2001 and 2003, or the Hot Oligonucleotide Ligation Assay (HOLA) [44] in samples collected in 2005 and 2007.

### Data analysis

Insecticide susceptibility data were analysed according to WHO criteria [41]: samples were defined as resistant if they showed less than 80% mortality; a mortality rate between 80-98% suggested reduced susceptibility but resistance needs to be confirmed, while mortality rates greater than 98% were indicative of complete susceptibility. The knockdown times for 50 and 95% of tested mosquitoes (KdT_50 _and KdT_95_) were estimated using a log-time probit model [45]. The KdT_50 _recorded from field-collected mosquitoes were compared with that of the *An. gambiae *Kisumu reference susceptible strain by estimates of KdT_50 _ratios (RR). Chi-square tests were used to compare the prevalence of M and S molecular forms of *An. gambiae *between the different study periods.

## Results

### Insecticide usage in study sites

More than 30 forms were filled in each study site, except in the Ipono timber yards where only one form was filled because chemical insecticides were not used for another purposes in this study area. Chemicals in use during year 2003 differed from one site to another. Table 1 summarises data collected from surveys conducted in the agricultural settings of Nkolondom and Mangoum. In these sites organophosphates (dimethoate, diazinon, chlorpyrifos-ethyl) and pyrethroids (lambda-cyhalothrin, cypermethrin and deltamethrin) were commonly used for crop protection. In addition, organochlorines (endosulfan and fipronil) and carbamates (carbofuran, methyl-parathion) were also used, especially in Nkolondom. However, large variability among gardeners in application dosages, the crop treatment cycles and the amounts of active ingredients used in these agricultural settings prevented quantitative analysis of the data.

**Table 1:** Insecticide usage in agricultural settings

Study site	Trade name (concentration)	Active ingredient	Class of insecticide and usage frequency (%)	Cultivated crops
Nkolondom	Bastion (100 g/kg)	Carbofuran	Carbamates (33)	Cabbage, parsley*
	Penncap.M (240 g/l), Sevin (850 g/kg	Methyl-parathion		Celery*, lettuce*,
	Callidim (400 g/l) Cyperdim (200 g/l)	Dimethoate	Organophosphorous (22)	Cabbage,
	Basudine (600 g/l)	Diazinon		Pepper*
	Thionex (500 g/l; 350 g/l), Thiodan (250 g/l 350 g/l)	Endosulfan	Organochlorines (20)	Tomato, spinach*
	Cypercal (12 g/l; 50 g/l; 100 g/l)	Cypermethrin	Pyrethroid (25)	Celery*, tomato, green bean, courgette, basil*
	Décis (12,5 g/l; 25 g/l; 60 g/l)	Deltamethrin		Eggplant
	Karate (2,5%; 5 g/l; 45 g/l)	Lambdacyhalothrin		Tomato, ginger

Mangoum	Thiodan (250 g/l; 350 g/l)	Endosulfan	Organochlorines (8)	Tomato*
	Regent (50 g/l)	Fipronil		Cabbage*
	Callidim (400 g/l), Cyperdim (200 g/l), Dimezyl (400 g/l), Planthoate (400 g/l)	Dimethoate	Organophosphorous (49)	Cabbage*, bean, melon*, watermelon*, tomato* lettuce, nightshade*
	Dursban (600 g/l), Pyriforce (600 g/l)	Chlorpyrifos-ethyl		Corn
	Pilori (15 g/l)	Lambdacyhalothrin	Pyrethroids (43)	Leek*, carrot
	Decis (25 g/l; 60 g/l; 12,5 g/l)	Deltamethrin		Green bean
	Méteor (400 g/l), Cigogne (50 g/l; 200 g/l), Cypercal (12 g/l; 50 g/l; 100 g/l), Cyperplant (100 g/l), Cythrine (25 g/l)	Cypermethrin		Green bean, corn, tomato*, carrot, leek*, nightshade*, potato

*: main cultivated crop

In Bonanloka (urban area), bomb sprays and coils containing pyrethroid insecticides with cyfluthrin, deltamethrin, lambda-cyhalothrin as common active ingredients, were mainly used for personal protection. In the timber yard in Ipono, pyrethroids (bifenthrin and cypermethrin) and organochlorines (lindane) were used in insecticide-fungicide mixtures to protect timber against xylophagous insects.

### Susceptibility to insecticides

**Figure 2:**
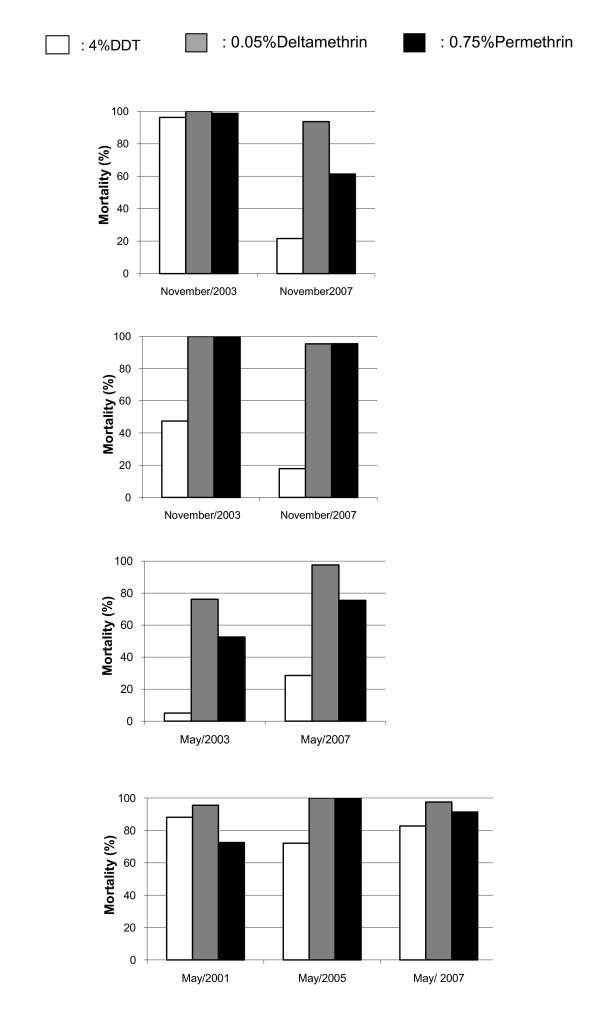
**Mortality of *Anopheles gambiae *24-hours post one-hour exposure to insecticide-impregnated papers at each study period**. (**A**): Ipono, (**B**): Nkolondom, (**C**): Mangoum, (**D**): Bonanloka.

Throughout the assays, the Kisumu strain of *An. gambiae *displayed mortality rates above 99% for the 3 insecticides tested and no significant variation was observed in knockdown times during the study periods. The kdT_50 _values were approximately 19.0; 9.5 and 8.5 minutes respectively for DDT, deltamethrin and permethrin. The corresponding kdT_95 _values were around 30, 25 and 15 minutes. In control groups of the Kisumu strain as well as that of the wild *An. gambiae *populations (unexposed mosquitoes) mortality rates 24 hours post-exposure were always below 5%. Mortality rates in field mosquito populations are shown in Figure 2 and their respective kdT_50 _and kdT_95 _are given in Table 2. Overall, the four *An. gambiae *populations showed different levels of resistance to the three insecticides from one year to another. In most cases, resistance was associated with an increase of knockdown times compared with the Kisumu susceptible strain, with a kdT_50 _ratio higher than 2 (Table 2).

**Table 2:** Knockdown time (kdT) for 50% and 95% of tested *Anopheles gambiae *s.l populations

Site	Insecticide	Period	N	kdT_50 _[CI_95_] (min)	kdT_95 _[CI_95_] (min)	RR	Resistant status
Ipono	4%DDT	Nov/2003	80	28.1 [22.4-31.9]	50.8 [45.0-62.7]	1.4	RC
	0.05%Deltamethrin		79	8.3 [5.4-10.9]	24.0 [18.1-38.9]	0.8	S
	0.75%Permethrin		79	11.5 [10.2-12.8]	44.3 [39.2-51.2]	1.3	S
	4%DDT	Nov/2007	87	>60	>60	>3	R
	0.05%Deltamethrin		95	13.7 [12.7-14.7]	34.1 [31.3-37.4]	1.4	RC
	0.75%Permethrin		88	37.3 [34.1-40.6]	>60	4.1	R

Nkolondom	4%DDT	Nov/2003	80	>60	>60	>3	R
	0.05%Deltamethrin		89	12.2 [3.7-18.8]	42.8 [32.7-61.0]	1.2	S
	0.75%Permethrin		86	12.6 [9.6-15.4]	47.1 [37.3-67.4]	1.4	S
	4%DDT	Nov/2007	84	>60	>60	>3	R
	0.05%Deltamethrin		89	20.1 [17.1-22.8]	45.8 [39. 1-58.4]	2.1	RC
	0.75%Permethrin		83	40.2 [37.6-42.8]	>60	4.5	RC

Mangoum	4%DDT	May/2003	80	>60	>60	>3	R
	0.05%Deltamethrin		84	24.1 [22.4-25.1]	39.2 [37.1-42.0]	2.5	R
	0.75%Permethrin		80	57.4 [52.3-68.0]	>60	6.6	R
	4%DDT	May/2007	91	>60	>60	>3	R
	0.05%Deltamethrin		84	19.4 [16.4-22.0]	48.3 [41.8-59.1]	2.0	RC
	0.75%Permethrin		106	54.2 [51.9-57.1]	>60	6.1	R

Bonanloka	4%DDT	May/2001	80	49.3 [41.8-58.7]	>60	2.6	RC
	0.05%Deltamethrin		86	22.4 [21.2-23.7]	34.8 [31.2-39.0]	2.4	RC
	0.75%Permethrin		91	16.3 [14.9-17.7]	25.7 [22.4-29.6]	1.7	R
	4%DDT	May/2005	80	51.6 [41.9-54.0]	>60	2.7	R
	0.05%Deltamethrin		83	8.3 [7.6-9.1]	18.6 [16.8-21.1]	0.8	S
	0.75%Permethrin		85	6.2 [3.6-8.5]	23.8 [18.0-37.2]	0.7	S
	4%DDT	May/2007	87	37.9 [36.5-39.5]	>60	1.9	RC
	0.05%Deltamethrin		84	12.8 [10.6-14.6]	24.1 [20.4-31.6]	1.3	RC
	0.75%Permethrin		88	15.2 [13.2-17.1]	35.3 [30.7-42.6]	1.7	RC

N: sample size; kdT_50_: knockdown time for 50% mosquitoes; kdT_95_: knockdown time for 95% mosquitoes; CI_95_: confidence interval at 95%; min: minutes; RR: resistance ratio (kdT_50 _of the tested population/kdT_50_of the Kisumu strain); S: susceptible; RC: resistance to be confirmed; R: resistant; Nov: November.

In the Ipono and Nkolondom populations, an increase of resistance to DDT and pyrethroids was observed from 2003 to 2007, as shown by an increase of knockdown times and a significant decrease in mortality (p < 0,05), particularly in the Ipono population (Table 2, Figure 2). Conversely, a decrease of resistance to DDT and pyrethroids was noted in the Mangoum population from 2003 to 2007; this decrease was mostly associated with significant increase of mortality rates (p < 0.05) in 2007 (Figure 2), although the knockdown times were slightly elevated. In Bonanloka, variations in resistance levels to DDT and pyrethroids were noted across the three surveys. DDT resistance was highest in 2005, whereas full susceptibility to pyrethroids was observed at this time. Reduced susceptibility to pyrethroids was observed in 2001 and 2007, however.

### Mosquito species, molecular forms and *kdr *frequencies

Samples of 40-50 specimens randomly drawn from control groups used for the susceptibility tests in each study site and period were identified molecularly to species and molecular form, and their genotype at the *kdr *locus was determined (Table 3). All mosquitoes tested belonged to the *An. gambiae *s.s species. The M and S molecular forms were found together in Ipono, Nkolondom and Bonanloka samples. The relative frequency of the M form which was 100% at Bonanloka in 2001 and 60.5% at Nkolondom in 2003 dropped to 58% and 4% in 2007, respectively (p < 0.0001). On the other hand, no significant difference was seen in the relative frequencies of both forms in Ipono between collections conducted in 2003 and 2007 (χ^2 ^= 0.17, p = 0.68). In Mangoum, only the S molecular form was found throughout the 2 study periods.

**Table 3:** Variations in molecular forms and *kdr*-alleles frequencies in tested *Anopheles gambiae *populations.

*An. gambiae*	Site	Year	N(%)	f(L1014S)	f(L1014F)
**M-form**	Ipono	2003	16 (33)	ND	0
		2005	22 (61)	0	0
		2007	19 (30)	0	0.10
	Nkolondom	2003	26 (60)	ND	0
		2005	0 (0)	-	-
		2007	2 (4)	0	0.50*
	Mangoum	2003	0 (0)	-	-
		2005	0 (0)	-	-
		2007	0 (0)	-	-
	Bonanloka	2001	30 (100)	ND	0
		2005	33 (63)	0	0
		2007	33 (58)	0	0.18

**S-form**	Ipono	2003	32 (67)	ND	0.04
		2005	14 (39)	0.03	0.25
		2007	45 (70)	0.18	0.62
	Nkolondom	2003	17 (39)	ND	0
		2005	64 (100)	0.03	0.59
		2007	45 (96)	0.02	0.92
	Mangoum	2003	51 (100)	ND	0.37
		2005	76 (100)	0.14	0.85
		2007	33 (100)	0.24	0.72
	Bonanloka	2001	0 (0)	-	ND
		2005	19 (37)	0	0.20
		2007	24 (42)	0	0.16

N: sample size; f(): frequency of the *kdr *alleles; *: 1 specimen out of the 2 tested carried the mutation; ND: not determined.

Both L1014F and L1014S *kdr *mutations were observed in three of the four *An. gambiae *populations tested, except in Bonanloka where only the L1014F mutation was recorded. Both mutations were found in individuals of the S molecular form while only the L1014F mutation was found in the M form, although at low frequencies (f < 0.2, Table 3) and in samples collected in 2007 only. In the three localities where the L1014S was detected in the S form, its frequency did not differ significantly between 2005 and 2007 (p > 0.07). However, a significant increase in the frequency of the L1014F mutation was detected in most of the S form populations between 2003 and 2007, especially in Nkolondom, where it rose from 0% in 2003 to 92% in 2007 (p < 0.0001).

### Resistance phenotype and *kdr *genotypes in M and S molecular forms from Ipono

To explore the distribution of resistance phenotypes and *kdr *genotypes within and between molecular forms of *An. gambiae*, we further analysed the samples from Ipono assayed in 2007, because of presence of both M and S molecular forms, and of both L1014F and L1014S *kdr *mutations in this site. A total of 214 *An. gambiae *s.s. specimens, randomly selected from control, dead or survivors to susceptibility tests (20-65 specimens per test) were successfully analysed. Among these mosquitoes, 46 (21.5%) were M form and 168 (78.5%) were S form (Table 4). No M form mosquito was identified within the group of survivors to insecticide exposure, suggesting complete susceptibility to the three compounds. Some dead specimens carried the L1014F allele at the heterozygous or homozygous state.

**Table 4:** Phenotypes and genotypes at codon 1014 of the vgsc of *Anopheles.	gambiae *collected in Ipono (December 2007).

Molecular form	Phenotype	Insecticide	N	Genotypes						f(Ser)	f(Phe)
						
				Leu-Leu	Leu-Phe	Leu-Ser	Ser-Ser	Phe-Phe	Phe-Ser		
**M-form**	Control	-	19	15	4	0	0	0	0	0	0.10
	Dead	4%DDT	8	6	2	0	0	0	0	0	0.12
		0.05%Deltamethrin	6	6	0	0	0	0	0	0	0
		0.75%Permethrin	13	8	2	0	0	3	0	0	0.31
	Survivor	4%DDT	0	-	-	-	-	-	-	-	-
		0.05%Deltamethrin	0	-	-	-	-	-	-	-	-
		0.75%Permethrin	0	-	-	-	-	-	-	-	-

**S-form**	Control	-	45	4	7	2	3	20	9	0.18	0.62
	Dead	4%DDT	11	2	4	3	0	1	1	0.18	0.41
		0.05%Deltamethrin	29	2	3	3	2	18	1	0.14	0.69
		0.75%Permethrin	15	2	4	1	1	4	3	0.20	0.50
	Survivor	4%DDT	29	0	1	0	0	27	1	0.01	0.96
		0.05%Deltamethrin	6	0	0	0	0	3	3	0.25	0.75
		0.75%Permethrin	33	0	0	0	0	24	9	0.14	0.86'

N: sample size; f(): frequency of the resistant alleles (L1014S = Ser and L1014F = Phe) in the mosquito pools; vgsc: voltage gated sodium channel

Among the 168 individuals of the S form, 26.8% belonged to control, 32.7% to dead and 40.5% to survivor samples. The two *kdr *mutations were present in control, dead or survivor mosquitoes, with a considerable number of homozygote (L1014F/L1014F) and heterozygote (L1014F/L1014S) individuals in each of the three mosquito classes. All possible genotype combinations were observed in the control and dead mosquitoes, whereas only three genotypes were recorded in surviving mosquitoes, most of which were either double heterozygotes (L1014F/L1014S) or homozygotes for the "West African" *kdr *mutation (L1014F/L1014F). No significant difference was seen in the frequency of the L1014S allele between dead and survivors to susceptibility tests, except with DDT where the frequency was lower in survivors (p < 0.001). The frequency of L1014F allele was significantly higher in mosquitoes surviving to DDT and permethrin (0.86-0.96) than in dead mosquitoes (0.41-0.50, P < 0.001). On the other hand, no significant difference was seen in L1014F frequency between dead and survivors to deltamethrin (P = 0.34). Overall, the frequencies of L1014F allele were higher in survivors than in the two other classes of mosquitoes.

## Discussion

The survey on insecticide usage provided evidence for intensive use of chemical insecticides both for agro-industry and for personal protection in South Cameroon. It is consistent with observations made by Akogbéto and colleagues [46] that the choice and usage of chemical compounds depends on cultivated crops and specification of devastating insects.

Susceptibility data presented above confirmed that wild *An. gambiae *s.s. from the study area show reduced susceptibility to DDT and pyrethroids [34,36,47]. The level of resistance however, varies from one year to another, according to the proportion of the resistant S form in the populations where the two molecular forms of *An. gambiae *were found together, especially in Nkolondom. In addition, the frequency of both *kdr *alleles increased in time within the S form in all sites where it was found. These results testify that the spread of the *kdr *alleles is an ongoing process in *An. gambiae *mosquito populations from Cameroon [36], as well as elsewhere in Central Africa [48,49].

The presence of the *kdr *mutations in the malaria vector *An. gambiae *has been monitored using a variety of molecular techniques [50]. In this study, molecular detection of East and West *kdr *genotypes were conducted using two PCR methods differing in their performance. Although the protocol assay used in 2003 [28] could not detected the 1014S allele, data collected in 2005 and 2007 using the protocol described by Lynd and colleagues [44] demonstrate the rapid invasion of the *kdr *mutation in the studied *An. gambiae *populations, especially in Ipono and Nkolondom.

Evidence of a strong correlation between resistance phenotype and *kdr *genotypes has been documented in previous studies [28,29]. However, results provided in this study, agree with the standpoint suggested by Brooke [50], arguing that *kdr *may act with certain co-factors that are thus far unidentified. This resistance mechanism could be multigenic, and the *kdr *genotype might not fully explain all the variance in the resistance phenotype [51]. However, this hypothesis does not rule out the causal relationship between the *kdr *genotype and susceptibility to DDT and pyrethroids [52].

The higher number of mutant homozygotes L1014F recorded in dead mosquitoes when exposed to 0.05% deltamethrin demonstrates that, *kdr *may be less efficient in providing resistance to deltamethrin than it is for DDT and permethrin resistance, as previously suggested [37,53,54]. Alternatively, the diagnostic dosage of deltamethrin may be very high and therefore killing resistant mosquitoes. Furthermore, other resistance mechanisms such as elevated oxidase, esterase or glutathione S-transferase activity may add to the *kdr *effect and increase resistance to DDT and permethrin in Ipono. Metabolic resistance has already been reported in *An. gambiae *and *An. arabiensis *populations from Cameroon [25,35,55], although specific analysis were not carried out to explore these mechanisms within each molecular form. Meanwhile, resistance levels such as that reported here seem to be strongly associated with the S molecular form. However, attention must also be paid to the M molecular form considering the increasing frequency of the L1014F *kdr *allele in this form [49].

The rise in frequency of both *kdr *mutations in *An. gambiae *is probably facilitated by the intensive use of the same insecticides in agriculture, wood industry and public health, resulting in ubiquitously high selection pressure for resistance in insects [56]. Reports from Cameroon as well as other African countries have mentioned the relation between public health or agricultural use of insecticides and the evolution of insecticide resistance in malaria vectors [34,55-57]. In addition to cotton or rice cultivation areas, market gardening areas, public health or personal protection, the current study put forward forest exploitation sites (timber yards) as potential zones of insecticide resistance emergence in *An. gambiae*.

This study further highlights the dynamics of *An. gambiae *susceptibility to insecticides in southern Cameroon. Biological, genetic and environmental factors may intervene in modulating susceptibility to insecticides.

## Conclusion

This study underlines the variability in the resistance level of *An. gambiae *s.s from southern Cameroon to the first-line insecticides currently used for its control. The current distribution of *kdr *mutations and ongoing trends for their geographical spread and rise in frequency need to be carefully considered. Such a situation is a real threat for malaria vector control strategies implemented in South Cameroon and calls for close collaboration between pest-management and vector control programmes towards the implementation of sustainable resistance management strategies.

## Competing interests

The authors declare that they have no competing interests.

## Authors' contributions

JE and FS conceive the study. JE, FS, RM, and PN designed the study protocol; JE, PN, MC, JCT, CKH and HPAA performed field work and bioassays; PN performed molecular analyses; PN and JE analyzed and interpreted the data; PN drafted the manuscript which was critically revised by JE, RM and FS. All the authors read and approved the final manuscript.

## Pre-publication history

The pre-publication history for this paper can be accessed here:

http://www.biomedcentral.com/1471-2334/9/163/prepub
